# Nitroxide Hormesis in Yeast: 4-Hydroxy-TEMPO Modulates Aging, and Cell Cycle

**DOI:** 10.3390/molecules31020376

**Published:** 2026-01-21

**Authors:** Mateusz Mołoń, Patrycja Kielar, Eliza Molestak, Agnieszka Mołoń, Ewelina Kuna, Marek Biesiadecki, Przemysław Grela, Alan González-Ibarra, Sabina Galiniak

**Affiliations:** 1 Faculty of Biology, Natural Protection, and Sustainable Development, University of Rzeszow, al. Tadeusza Rejtana 16C, 35-959 Rzeszów, Poland; ekuna@ur.edu.pl; 2 Faculty of Medicine, University of Rzeszów, al. Tadeusza Rejtana 16C, 35-959 Rzeszów, Poland; pkielar@ur.edu.pl (P.K.); agmolon@ur.edu.pl (A.M.); mbiesiadecki@ur.edu.pl (M.B.); 3 Department of Molecular Biology, Institute of Biological Sciences, Maria Curie-Skłodowska University, Akademicka 19, 20-033 Lublin, Poland; eliza.molestak@mail.umcs.pl (E.M.); przemyslaw.grela@mail.umcs.pl (P.G.); quimicalex23@gmail.com (A.G.-I.)

**Keywords:** aging, cell cycle, RNA-Seq, ribosome, yeast, 4-hydroxy-TEMPO

## Abstract

4-hydroxy-TEMPO is a water-soluble nitroxide radical with potent antioxidant and redox-modulating properties. Its small molecular weight and membrane permeability enable it to act as a superoxide dismutase mimetic, efficiently scavenging reactive oxygen species and mitigating oxidative damage. In this study, we investigated the physiological and transcriptomic effects of 4-hydroxy-TEMPO in *Saccharomyces cerevisiae*, using wild-type and mutant strains deficient in key redox and DNA repair pathways (*sod1Δ*, *sod2Δ*, *yap1Δ*, *rad52Δ*). RNA-Seq analysis revealed widespread transcriptional reprogramming. Treatment with 4-hydroxy-TEMPO impaired cell growth, induced accumulation of cells with 1C (G1 phase) DNA content, and modulated chronological aging in a strain-dependent manner. Notably, low concentrations delayed aging in wild-type, *yap1Δ*, and *rad52Δ* strains, while accelerating it in *sod1Δ* mutants, consistent with a hormetic response. Unlike TEMPO, 4-hydroxy-TEMPO exhibited markedly reduced translational toxicity, preserved polysome structure at high doses, and triggered a non-canonical, redox-dependent transcriptional program characterized by induction of stress-response genes together with unexpected up-regulation of multiple ribosomal protein genes. This was accompanied by a biphasic, genotype-specific hormetic response and a measurable genoprotective effect. RT-qPCR confirmed key transcriptional changes, linking transcriptome remodeling to functional outcomes.

## 1. Introduction

4-Hydroxy-2,2,6,6-tetramethylpiperidine-1-oxyl, commonly known as Tempol, is a heterocyclic nitroxide derivative with notable antioxidant properties. Its enhanced water solubility is attributed to the hydroxyl group at the C-4 position, which also imparts distinct biological characteristics compared to its analog, TEMPO [1]. As a stable free radical, 4-hydroxy-TEMPO functions effectively as both a catalyst and an oxidizing agent. Its low molecular weight (172 Da) allows for rapid diffusion across cellular membranes [2,3,4,5].

Functionally, 4-hydroxy-TEMPO acts as a superoxide dismutase (SOD) mimetic, capable of crossing membranes and accumulating in the cytoplasm—a feature that distinguishes it from recombinant SOD. Numerous studies have shown that 4-hydroxy-TEMPO reduces the activity of superoxide anions (O_2_^•−^) [6,7]. Its efficiency is considered comparable to both SOD and catalase. The compound primarily safeguards by neutralizing the creation of hydroxyl radicals and lessening injury caused by hydrogen peroxide [4,8]. Additional evidence suggests that 4-hydroxy-TEMPO may also influence membrane fluidity and permeability [4].

Given that oxidative stress plays a central role in the pathogenesis of many diseases, 4-hydroxy-TEMPO and related nitroxides have garnered attention as potential therapeutic agents [9]. Studies have demonstrated their ability to reduce lipid peroxidation, protein oxidation, and DNA damage under conditions of elevated ROS generation [10,11,12,13,14]. For example, Jahan et al. reported that 4-hydroxy-TEMPO significantly lowered oxidative stress markers, such as malondialdehyde and advanced protein oxidation products, in tissues of rats treated with carbon tetrachloride [15]. Similarly, Rossetto et al. identified 4-hydroxy-TEMPO as a promising candidate for prostate cancer therapy due to its antioxidant activity and low toxicity [16]. Moreover (4-hydroxy-TEMPO also exhibits neuroprotective effects, primarily by limiting mitochondrial dysfunction. In differentiated human neuroblastoma cells (SH-SY5Y) exposed to 6-hydroxydopamine, 4-hydroxy-TEMPO reduced mitochondrial superoxide production and lipid peroxidation, suppressed heme oxygenase-1 induction and NF-κB activation, mitigated cytotoxicity, and partially normalized the expression of dopamine D2 receptor isoforms and SOD1 [17]. Although TEMPO and 4-hydroxy-TEMPO are structurally related nitroxides, they exhibit markedly different physicochemical and biological properties due to the presence of a hydroxyl group at the C-4 position in 4-hydroxy-TEMPO. Our previous study focused exclusively on TEMPO and demonstrated its pronounced translational toxicity and impact on ribosome biogenesis; however, those findings cannot be extrapolated to 4-hydroxy-TEMPO [18].

In this study, we aimed to investigate—for the first time—the effects of 4-hydroxy-TEMPO on physiological parameters and chronological aging in wild-type (WT) yeast and isogenic mutants lacking *SOD1*, *SOD2*, *RAD52*, and *YAP1*. RNA-Seq analysis revealed that this nitroxide compound induces widespread transcriptional changes, disrupting key metabolic and stress response pathways. We hypothesized that 4-hydroxy-TEMPO modulates yeast aging and genome stability through redox-dependent control of ribosomal and DNA repair pathways.

## 2. Results

### 2.1. 4-Hydroxy-TEMPO Inhibits Yeast Growth and Clonogenicity

4-hydroxy-TEMPO significantly impairs yeast cell proliferation. To investigate this effect in vivo, we employed four haploid deletion mutants—*sod1Δ*, *sod2Δ*, *rad52Δ*, and *yap1Δ*—all derived from the BY4741 genetic background. The *sod1Δ* and *sod2Δ* strains lack cytosolic and mitochondrial SOD activity, respectively, enzymes that play a key role in detoxifying superoxide radicals and protecting cells from oxidative stress. The *rad52Δ* mutant is deficient in a protein essential for homologous recombination and the repair of DNA double-strand breaks, contributing to genome stability. Meanwhile, *YAP1* encodes a transcription factor that regulates the cellular response to oxidative stress; its deletion renders cells hypersensitive to redox imbalance.

**Figure 1:**
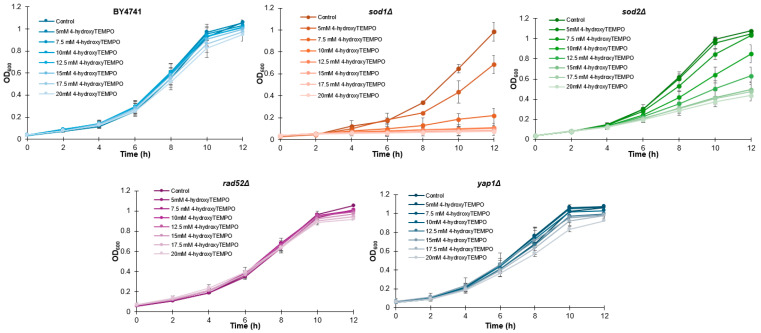
Growth kinetics of the haploid WT yeast strain BY4741 and its isogenic deletion mutants (*sod1Δ*, *sod2Δ*, *rad52Δ*, and *yap1Δ*) were compared following treatment with 4-hydroxy-TEMPO. Optical density at 600 nm (OD_600_) was measured at multiple time points over a 12 h period. The data represent biological replicates from three independent cultures, with SD values equal to or smaller than the symbol size. Each condition was validated in three independent experiments conducted on separate days.

To determine whether 4-hydroxy-TEMPO affects yeast growth, we first monitored the proliferation of all strains over a 12 h period. Growth curves were generated for each strain treated with increasing concentrations of 4-hydroxy-TEMPO (5, 7.5, 10, 12.5, 15, 17.5, and 20 mM), alongside untreated controls. As shown in Figure 1, nitroxide supplementation led to a dose-dependent inhibition of growth across all strains, with *sod1Δ* and *sod2Δ* exhibiting the most pronounced sensitivity.

**Figure 2:**
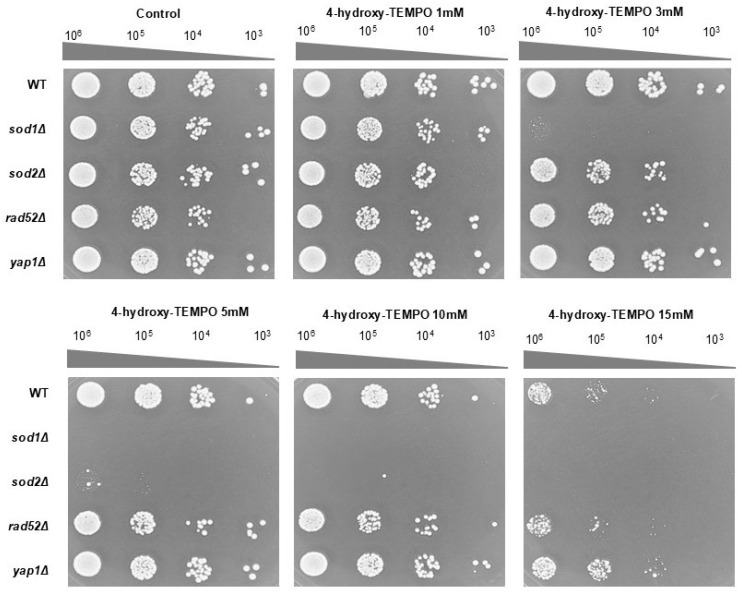
The growth response of the BY4741 WT strain and its isogenic deletion mutants (*sod1Δ*, *sod2Δ*, *rad52Δ*, and *yap1Δ*) was evaluated under increasing concentrations of 4-hydroxy-TEMPO. Growth on untreated YPD agar plates served as the control condition. Representative results from three independent experiments are shown.

To further validate these findings, we performed a spot assay on rich solid medium following 48 h of incubation. At 3 mM, 4-hydroxy-TEMPO markedly suppressed the growth of *sod1Δ*, while other strains showed minimal response. At 5 mM, growth of both *sod1Δ* and *sod2Δ* was completely inhibited. In contrast, the WT and *yap1Δ* strains displayed the highest resistance to nitroxide treatment (Figure 2). These findings demonstrate that both prolonged incubation and higher concentrations of 4-hydroxy-TEMPO exert an inhibitory effect on the growth of the tested yeast strains. Notably, the compound significantly reduced the clonogenic potential of *sod1Δ*, *sod2Δ*, and *rad52Δ* mutants, indicating increased sensitivity in strains deficient in oxidative stress response and DNA repair. However, these results alone do not fully account for the cytotoxicity of the nitroxide. To further investigate its impact on cell viability, a survival assay was conducted using the fluorescent dye propidium iodide. Fluorescence microscopy revealed no evidence of cell death following 12 h of treatment with 4-hydroxy-TEMPO. All strains exhibited survival rates comparable to untreated controls, with viability remaining at 100%.

To further investigate the impact of 4-hydroxy-TEMPO on yeast cell behavior over time, microscopic observations were performed using all analyzed strains under increasing concentrations of the compound (0, 5, 10, 20 mM). As shown in Supplementary Dataset S1, cell clonogenicity was monitored at 0, 3, 6, 12, and 24 h. In untreated control cells, a progressive increase in cell clustering was observed, culminating in dense colony by 24 h. Treatment with 5 mM 4-hydroxy-TEMPO resulted in visibly reduced clonogenicity, with smaller clusters forming over time, mainly in *sod1D* and *sod2D* strains. This inhibitory effect was more pronounced at 10 mM, where cell growth was inhibited in WT, *rad52D* and *yap1D*. At the highest concentration tested (20 mM), cells remained dispersed throughout the 24 h period, indicating a strong suppression of growth and cell cycle halt.

**Figure 3:**
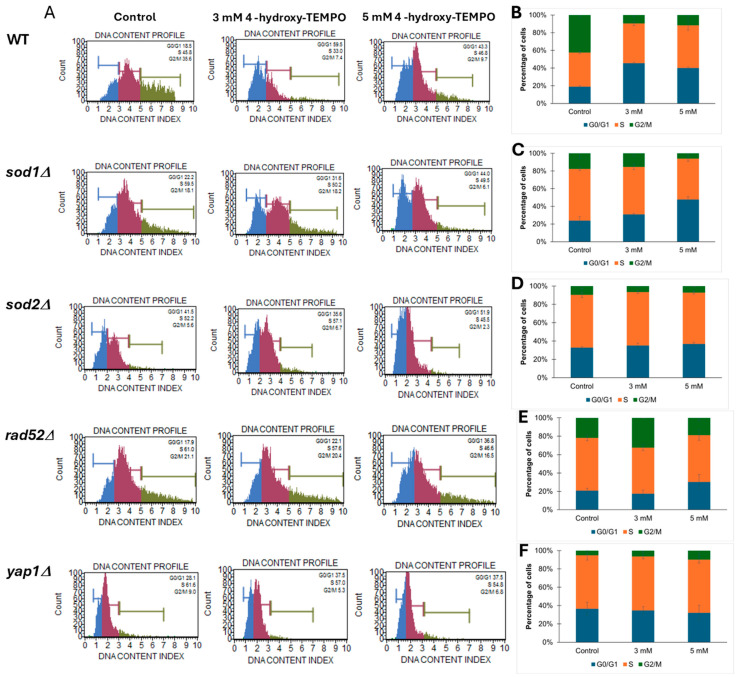
Cell cycle analysis of yeast strains treated with 4-hydroxy-TEMPO. (**A**) Representative DNA content histograms obtained from Muse™ Cell Analyzer showing cell cycle phase distribution in yeast strains under control and treatment conditions. (**B**–**F**) Quantitative analysis of cell cycle phase percentages (G0/G1, S, G2/M) in BY4741 WT, *sod1Δ*, *sod2Δ*, *rad52Δ*, and *yap1Δ* strains, respectively, following treatment with 3 mM and 5 mM 4-hydroxy-TEMPO for 2 h. Data are presented as mean ± SD from three independent experiments.

To further investigate whether the growth curve alterations observed upon 4-hydroxy-TEMPO treatment were associated with changes in cell cycle dynamics, we performed cell cycle analysis on WT and mutant yeast strains (*sod1Δ*, *sod2Δ*, *rad52Δ*, *yap1Δ*). As shown in Figure 3, panel A displays representative DNA content histograms, while panels B–F present quantitative distributions of cells in G1, S, and G2/M phases under control conditions and following treatment with 3 mM or 5 mM 4-hydroxy-TEMPO. The nitroxide compound induced cell cycle disturbances primarily at the G1 and G2/M phases. In WT cells, we observed an accumulation of cells in G1 phase accompanied by a reduction in the G2/M population, suggesting a delay in cell cycle progression. A similar pattern was evident in *sod1Δ* cells, which showed pronounced G2/M accumulation, particularly at 5 mM, indicating increased sensitivity to oxidative stress due to impaired superoxide dismutase activity (Figure 3A,C). The *sod2Δ* strain exhibited comparable but less pronounced changes (Figure 3A,D). In *rad52Δ* cells, which lack a key DNA repair protein, treatment led to a marked increase in the accumulation of cells with 2C (G2/M) DNA content, consistent with replication stress or activation of DNA damage checkpoints (Figure 3A,E). *yap1Δ* mutants, deficient in oxidative stress response regulation, also accumulated in G2/M phase, underscoring the role of Yap1 in maintaining cell cycle fidelity under redox imbalance (Figure 3A,F). Notably, these cell cycle alterations were most evident at the higher concentration of 4-hydroxy-TEMPO, suggesting a dose-dependent effect. Overall, our findings demonstrate that 4-hydroxy-TEMPO disrupts cell cycle progression, with mutant strains—particularly *sod1Δ*—exhibiting heightened sensitivity due to compromised oxidative stress defense mechanisms.

### 2.2. 4-Hydroxy-TEMPO Modulates Chronological Lifespan in a Strain-Dependent Manner

**Figure 4:**
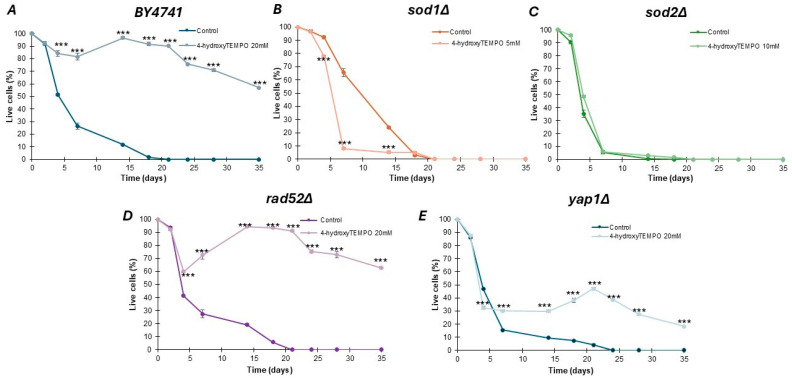
Chronological lifespan analysis of haploid *S. cerevisiae* strain BY4741 (**A**) and isogenic single mutants *sod1Δ* (**B**), *sod2Δ* (**C**), *rad52Δ* (**D**), *yap1Δ* (**E**), either untreated or exposed to 4-hydroxy-TEMPO. Lifespan was monitored over time, and survival rates were compared to untreated controls. Statistical significance was determined using one-way ANOVA followed by Dunnett’s post hoc test (*** *p* < 0.001). Error bars represent SD from three independent experiments.

Identifying compounds that can delay cellular aging remains a central objective in contemporary biogerontology. Given the documented antioxidant properties of 4-hydroxy-TEMPO in both in vitro and in vivo systems, we investigated its potential role in promoting longevity. Chronological lifespan was assessed by monitoring the survival of yeast populations in the stationary phase at defined time intervals. As illustrated in Figure 4, supplementation with 4-hydroxy-TEMPO exerted a beneficial effect on the viability of nearly all tested strains. These findings suggest that the nitroxide compound may enhance cellular resilience during aging, potentially through mechanisms linked to oxidative stress mitigation. In this study, various concentrations of 4-hydroxy-TEMPO were applied to ensure growth-inhibitory effects across all tested yeast strains. As shown in Figure 4A,D,E, treatment with the nitroxide significantly extended the chronological lifespan of post-mitotic cells in WT *rad52Δ*, and *yap1Δ* strains (*p* < 0.001). Between days 15 and 20 of the experiment, we observed a late-life regrowth-like feature (a transient increase in the viability readout), consistent with the gasping/adaptive regrowth phenomenon described in chronological aging assays [19,20,21]. However, CLS measurements alone do not discriminate between true population regrowth and reversible growth arrest. Notably, by day 35, cultures of WT and *rad52Δ* maintained approximately 60% viability, while *yap1Δ* showed a reduced survival rate of around 20%. Interestingly, the effect of 4-hydroxy-TEMPO was markedly different in mutants lacking SOD activity. The highest mortality was recorded in *sod1Δ* cells, with a dramatic 90% loss of viability by day 7 (*p* < 0.001) (Figure 4B). Interestingly, the nitroxide had no statistically significant impact on the survival of *sod2Δ* cells (Figure 4C).

### 2.3. 4-Hydroxy-TEMPO Alters Redox State and Metabolic Activity

The metabolic activity of yeast cells was evaluated using the FUN-1 fluorescent probe, which provides insight into both cell viability and metabolic function. FUN-1 passively diffuses into fungal cells, emitting green fluorescence. In metabolically active cells, the dye undergoes intracellular processing that shifts the fluorescence to red, whereas dead cells retain a light green signal.

**Figure 5:**
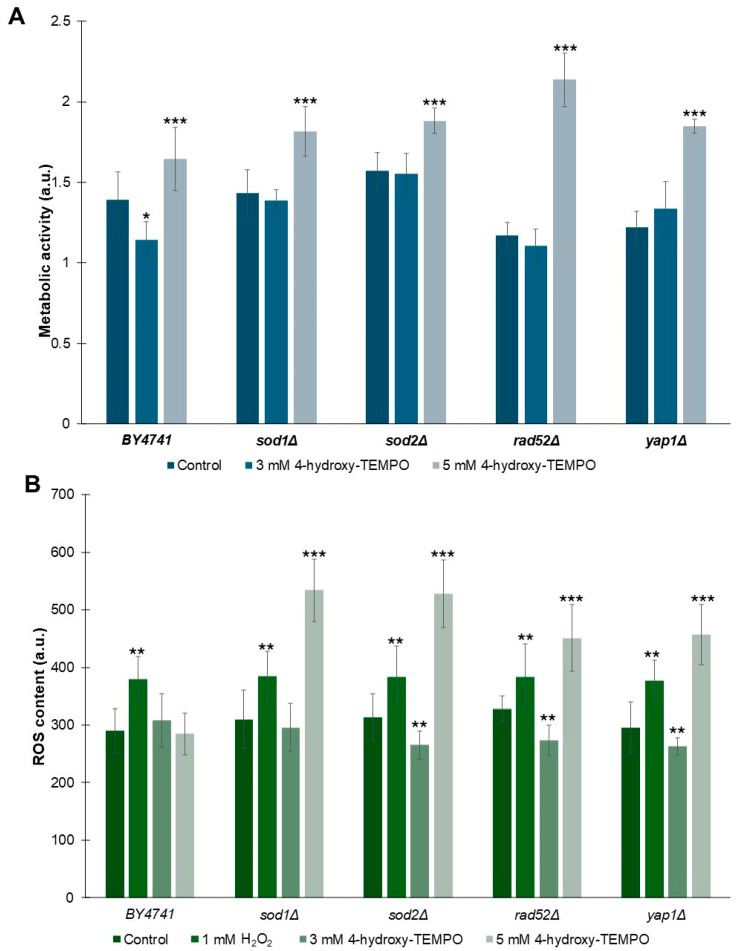
Influence of 4-hydroxy-TEMPO on metabolic activity and superoxide anion production in yeast cells. (**A**) Metabolic activity of BY4741 and haploid isogenic deletion mutants exposed to 3 mM or 5 mM 4-hydroxy-TEMPO for 2 h was assessed using the FUN-1 fluorescent probe. Data are expressed as mean ± standard deviation from three independent biological replicates. (**B**) Superoxide anion levels were quantified using dihydroethidine (DHE) fluorescence in yeast cells cultured in liquid medium supplemented with 4-hydroxy-TEMPO. DHE fluorescence reflects the signal from oxidized DHE products. Hydrogen peroxide (1 mM H_2_O_2_) was used as a positive control for oxidative stress induction. Data are presented as mean ± standard deviation from three independent replicates. Statistical significance was determined using one-way ANOVA followed by Dunnett’s post hoc test, with comparisons made against untreated controls (* *p* < 0.05; ** *p* < 0.01; *** *p* < 0.001).

As shown in Figure 5A, treatment with 5 mM 4-hydroxy-TEMPO significantly enhanced metabolic activity across all tested strains (*p* < 0.001). In contrast, exposure to 3 mM 4-hydroxy-TEMPO did not alter metabolic activity in *sod1Δ*, *sod2Δ*, or *rad52Δ* strains. Interestingly, WT cells exhibited a modest but statistically significant reduction in metabolic activity at this concentration compared to untreated controls (*p* < 0.05). Notably, despite this decrease in WT, the 3 mM nitroxide treatment led to a slightly increase in metabolic activity in *yap1Δ* strains, suggesting a strain-specific response to submaximal concentrations of 4-hydroxy-TEMPO. To evaluate whether nitroxide treatment influences ROS generation in yeast cells, we exposed cultures to 4-hydroxy-TEMPO at concentrations of 3 mM and 5 mM for 2 h. As a positive control, cells were treated with 1 mM hydrogen peroxide for 2 h. As shown in Figure 5B, hydrogen peroxide induced a statistically significant increase in ROS levels across all analyzed strains (*p* < 0.01), confirming its pro-oxidative effect. Interestingly, we observed a concentration-dependent biphasic response to 4-hydroxy-TEMPO. Treatment with 3 mM 4-hydroxy-TEMPO resulted in a reduction in intracellular ROS in almost all mutant strains, with no significant change in the WT and *sod1Δ*. In contrast, exposure to 5 mM 4-hydroxy-TEMPO led to a significant increase in ROS levels in all mutant strains (*p* < 0.001), while paradoxically slightly reducing ROS in WT cells. This dual effect suggests that 4-hydroxy-TEMPO may act as both an antioxidant and a pro-oxidant depending on concentration and genetic background, a phenomenon consistent with redox-modulating compounds.

### 2.4. RNA-Seq Reveals Extensive Remodeling of Translation-Related and Stress-Response Pathways

RNA sequencing (RNA-Seq) has become a cornerstone of modern transcriptomic analysis, offering unparalleled sensitivity, accuracy, and genome-wide coverage. Unlike traditional gene expression methods, RNA-Seq enables the simultaneous quantification of all transcripts, detection of novel isoforms, and identification of subtle changes in gene regulation. This technology allows researchers to uncover complex biological responses, perform functional enrichment analyses, and prioritize candidate genes for further investigation based on statistically robust data. Principal component analysis (PCA) of normalized RNA-Seq counts showed tight clustering of the three biological replicates in each condition and a clear separation between control and 4-hydroxy-TEMPO–treated samples along PC1, which explained 90% of the total variance. This indicates a strong and highly reproducible transcriptional response to 4-hydroxy-TEMPO and argues against batch- or normalization-related artifacts.

**Figure 6:**
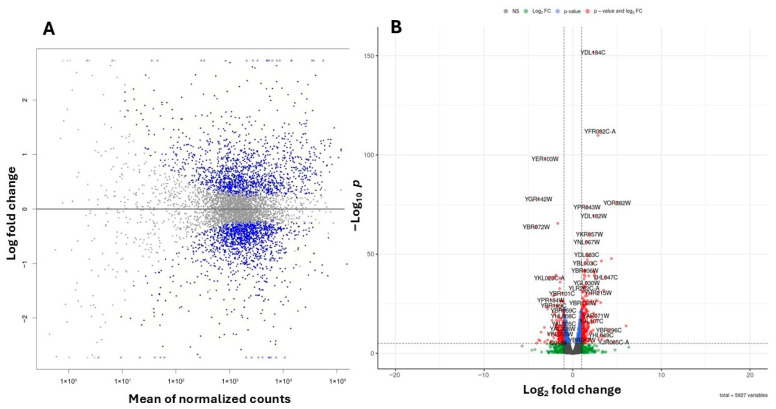
Summary plots of differential gene expression. (**A**) MA plot illustrating the relationship between the logarithmic fold change and the normalized average expression for each gene. Each point represents an individual gene. Negative values indicate lower expression in 4-hydroxy-TEMPO–treated BY4741 relative to untreated BY4741, while positive values indicate higher expression in treated BY4741 relative to untreated BY4741. Genes with FDR < 0.05 are marked in blue. (**B**) Volcano plot illustrating the differential expression of genes between untreated BY4741 cells and those treated with 20 mM 4-hydroxy-TEMPO for 2 h. Genes with FDR < 0.05 are marked in blue, those with an absolute log_2_ fold change greater than 1 are shown in green, while genes meeting both criteria are highlighted in red. Genes not fulfilling these thresholds are depicted in gray.

To investigate the molecular basis of these phenotypic changes, we performed RNA-Seq analysis on WT yeast cells treated with 20 mM 4-hydroxy-TEMPO for 2 h, compared to untreated controls. Transcriptome profiling was performed using high-throughput RNA sequencing, and differential gene expression was identified by applying a False Discovery Rate (FDR) threshold of <0.05 to account for multiple hypothesis testing. We analyzed gene expression profiles in cells treated with 20 mM 4-hydroxy-TEMPO and compared them to untreated controls, revealing distinct differences in cellular responses (Figure 6A,B). Differential expression analysis identified 1391 upregulated and 1365 downregulated genes in response to nitroxide treatment (Figure 6A,B).

Out of 6191 annotated yeast genes, 2756 (44.52%) were significantly differentially expressed (*p* < 0.05). Among the genes exhibiting the most pronounced changes in expression, ribosomal genes from both the large and small subunits are particularly notable. These include YDL184C, YFR032C-A, YPR043W, YGL147C (large subunit) and YKR057W, YMR143W, YGR118W (small subunit). Additionally, genes involved in lipid metabolism (YGL055W), protein folding and stress response (YER103W, YDR258C, YBR072W), and vacuolar protein sorting (YBR185C, YOR382W) also showed significant differential expression. (Figure 6B).

**Figure 7:**
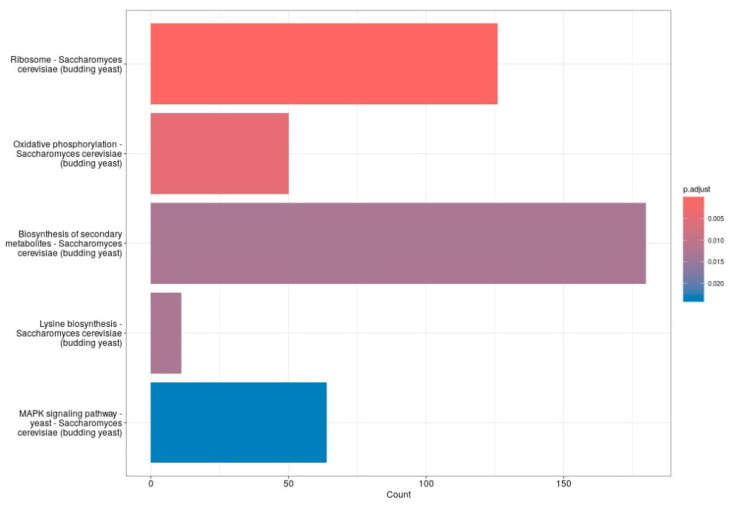
The bar plots display the top five enriched functions. The *X*-axis represents the number of genes in each gene set, while the *Y*-axis lists the gene set functions. Bar color indicates the adjusted *p*-value, with red showing the most significant results and blue indicating the least significant.

Pathway enrichment analysis of differentially expressed genes revealed significant modulation of several biological processes in *S. cerevisiae* following treatment with 20 mM 4-hydroxy-TEMPO. Notably, genes associated with ribosomal function—encompassing both large and small subunits—were among the most prominently affected. Consistent with these findings, KEGG pathway analysis highlighted enrichment in pathways such as ribosome, oxidative phosphorylation, lysine biosynthesis, and MAPK signaling, with the biosynthesis of secondary metabolites pathway showing the highest gene count and statistical significance (Figure 7). These results suggest that nitroxide treatment elicits a broad cellular response, affecting both core metabolic functions and stress adaptation mechanisms. We consider the observed alterations in the MAPK signaling pathway to be particularly significant. This evolutionarily conserved cascade plays a pivotal role in coordinating cellular responses to environmental and physiological stressors. In budding yeast, MAPK pathways regulate essential processes including cell cycle progression, differentiation, apoptosis, and adaptation to oxidative stress. The enrichment of MAPK-related genes following nitroxide exposure suggests activation of stress-responsive signaling networks, potentially contributing to the modulation of protein homeostasis, survival mechanisms, and broader transcriptional reprogramming.

**Figure 8:**
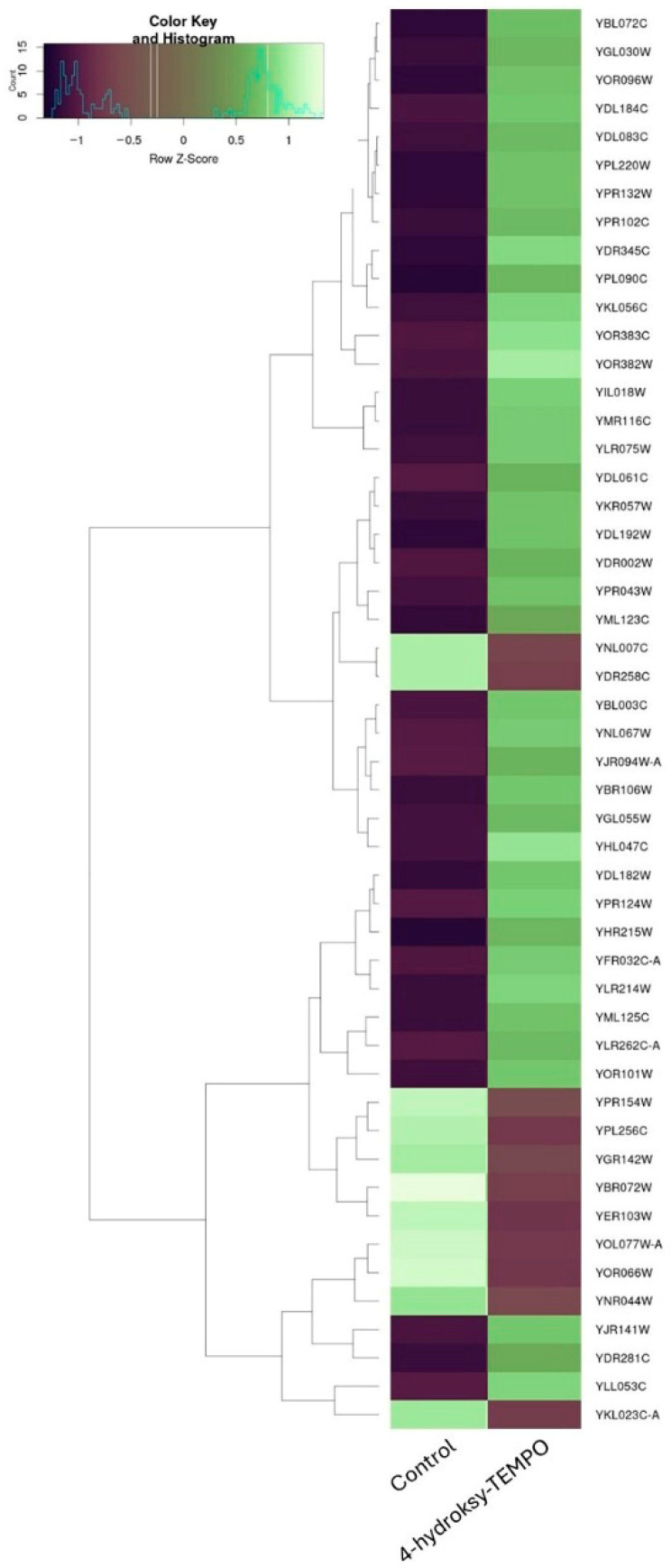
Hierarchical clustering heatmap of gene expression changes in *S. cerevisiae* following 4-hydroxy-TEMPO treatment. The heatmap displays the expression profiles of the most significantly altered genes under control and 4-hydroxy-TEMPO-treated conditions.

Treatment of *S. cerevisiae* with 4-hydroxy-TEMPO induced substantial transcriptional reprogramming, affecting a diverse array of functional gene categories. To further illustrate the extent and directionality of these changes, we constructed a heatmap representing the most significantly affected genes. This visualization highlights comprehensive shifts in gene expression—encompassing both up- and down-regulated transcripts—that reflect coordinated changes in cellular stress responses, metabolic reprogramming, and translational regulation (Figure 8). A prominent group of differentially expressed genes includes those encoding ribosomal proteins, suggesting a strong impact on the translational machinery. Both large subunit ribosomal protein genes (e.g., *YGL135W*, *YLR075W*, *YGL147C*, *YLR075W*, *YPR102C*, *YFR032C-A*, *YGL030W*, *YDL184C*, *YPR043W*, *YNL301C*) and small subunit components (e.g., *YLR344W*, *YOR096W*, *YER102W*, *YMR143W*, *YKR057W*, *YGR118W*, *YFR032C-B*) were affected, along with *YMR116C*, a ribosome-associated protein involved in translation regulation. These changes may reflect a cellular strategy to modulate protein synthesis under oxidative stress conditions (Supplementary Dataset S1).

Genes associated with stress response were also notably upregulated, including molecular chaperones *YBR072W*, *YDR258C*, *YER103W*, *YNL007C*, as well as *YBR185C*, which is involved in protein sorting during stress.

Metabolic and transport-related genes showed substantial modulation. YGL055W, encoding a fatty acid desaturase, indicates alterations in lipid metabolism. Iron and copper homeostasis appeared disrupted, as evidenced by changes in YOR382W, YOR383C, *YLR214W*, *YHL040C*, and *YPR124W*. Phosphate transporters YML123C and YHR215W were also upregulated, alongside *YDR345C*, a hexose transporter. Additional transport-related genes such as *YDR002W* and *YBR108W* suggest broader effects on membrane dynamics and nutrient uptake. The complete differential expression table is provided as Supplementary Dataset S1.

Cell cycle and growth regulation genes were among those altered, including *YPL256C*, *YDL192W*, and *YOR101W*, indicating potential interference with cell cycle progression and proliferation.

Transcriptional and chromatin-related genes such as YBL003C and YJR140C were differentially expressed, suggesting epigenetic remodeling or transcriptional reprogramming in response to oxidative stress.

Mitochondrial function also appeared affected, with changes in *YOR292C* and mitochondrial assembly factors *YBR181C* and *YIL106W*, pointing to possible mitochondrial stress or altered bioenergetics. The amino acid biosynthesis gene YLR303W, involved in lysine production, was modulated, indicating metabolic adaptation to stress conditions. Finally, several poorly annotated or uncharacterized genes (*YKL023C-A*, *YLL053C*, *YDL106C*, *YNL075W*, *YNR044W*) were significantly altered, potentially representing novel components of the oxidative stress response. Their roles remain to be elucidated in future studies.

Overall, the transcriptional profile induced by 4-hydroxy-TEMPO highlights its multifaceted impact on yeast physiology, affecting translation, stress response, metabolism, cell cycle regulation, and mitochondrial function. Taken together, these findings suggest that 4-hydroxy-TEMPO induces a survival-oriented physiological state, characterized by reduced proliferative and metabolic activity together with activation of stress-responsive programs. Such transcriptional reprogramming is consistent with a priming or conditioning effect that enhances cellular resilience to subsequent insults, rather than a direct stimulation of DNA repair pathways.

Treatment of WT yeast cells with 4-hydroxy-TEMPO resulted in pronounced transcriptional reprogramming, as revealed by Gene Ontology (GO) enrichment analysis. The most significantly upregulated categories were associated with protein synthesis and ribosomal structure. Notably, genes involved in translation (GO: 0006412), cytoplasmic translation (GO:0002181), and structural constituents of the ribosome (GO:0003735) showed strong enrichment, with over 100 genes upregulated in each category. Additional ribosome-related terms, including ribosomal subunit (GO:0044391), cytosolic ribosome (GO:0022626), and large ribosomal subunit (GO:0015934), were also prominently activated, indicating a robust stimulation of the translational machinery (Table 1).

In parallel, genes linked to oxidoreductase activity (GO:0016491) and antioxidant activity (GO:0016209) were upregulated, reflecting an enhanced cellular response to oxidative stress. This is consistent with the known redox-modulating properties of 4-hydroxy-TEMPO. Furthermore, increased expression of genes associated with plasma membrane (GO:0005886) and monovalent cation transport (GO:0006812) suggests alterations in membrane dynamics and ion homeostasis.

Conversely, several biological processes were significantly downregulated. Metabolic pathways such as generation of precursor metabolites and energy (GO:0006091) and aerobic respiration (GO:0009060) were suppressed, indicating a shift away from energy production. Additionally, key regulators of the cell cycle were downregulated, including genes involved in negative (GO:0010948) and positive regulation of cell cycle processes (GO:0090068), as well as cell death (GO:0008219), suggesting a dampening of proliferative and apoptotic signaling. These changes likely reflect a cellular adaptation to oxidative stress, prioritizing translational and protective mechanisms over growth and energy metabolism.

**Figure 9:**
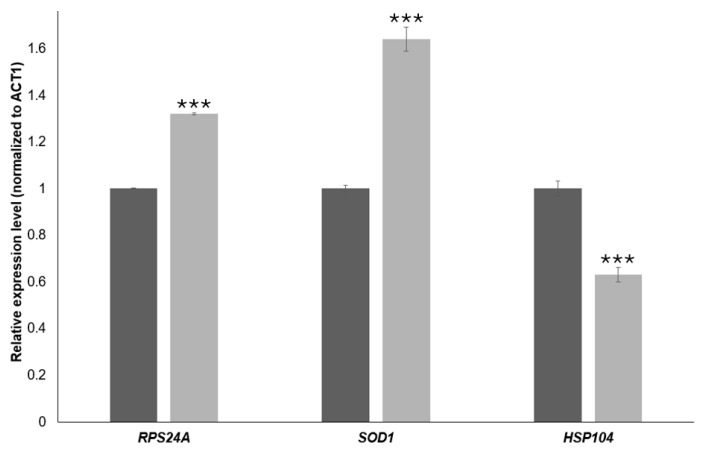
RT-qPCR validation of gene expression changes induced by 4-hydroxy-TEMPO. Relative transcript levels of *RPS24A*, *SOD1*, and *HSP104* were quantified by real-time PCR and normalized to *ACT1* as the internal reference gene. Expression values were calculated using the 2^−ΔΔCt^ method and are presented as relative fold change compared with untreated control cells (dark bars). Light bars represent BY4741 cells treated with 4-hydroxy-TEMPO (20 mM for 2 h) under the RNA-Seq experimental conditions. Statistical significance was determined using one-way ANOVA followed by Dunnett’s post hoc test, with comparisons made against untreated controls (*** *p* < 0.001). Data are shown as mean ± SD from three independent biological replicates.

To validate the RNA-Seq data, we quantified the expression of three representative genes by RT-qPCR in BY4741 cells treated with 20 mM 4-hydroxy-TEMPO for 2 h. Transcript levels were normalized to *ACT1* and analyzed using the 2^−ΔΔCt^ method. As shown in Figure 9, *RPS24A* and *SOD1* were significantly up-regulated upon 4-hydroxy-TEMPO treatment (≈1.3-fold and ≈1.6-fold increase, respectively; ** *p* < 0.001 vs. untreated control), whereas *HSP104* expression was markedly reduced to ~60% of control levels (** *p* < 0.001). These qPCR results are fully consistent with the RNA-Seq data and confirm that 4-hydroxy-TEMPO induces the up-regulation of a ribosomal protein gene and an antioxidant defense gene while concomitantly down-regulating the disaggregase chaperone HSP104, supporting the notion of a non-canonical, redox-driven stress response.

**Figure 10:**
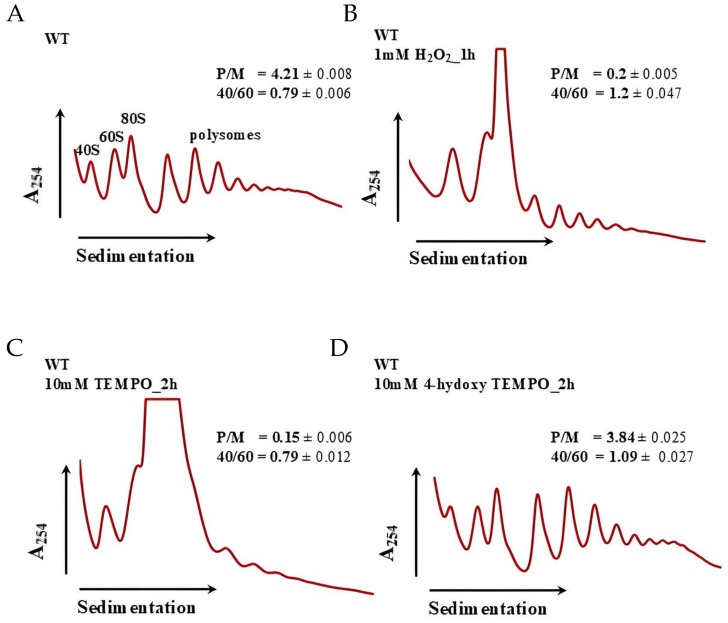
Polysome profile analysis of *S. cerevisiae* cells untreated (**A**), treated with 1 mM H_2_O_2_ for 1 h (**B**), treated with 10 mM TEMPO for 2 h (**C**) and treated with 10 mM 4-hydroxy-TEMPO for 2 h (**D**). Polysome profiles were generated to assess the impact of 4-hydroxy-TEMPO on ribosome biogenesis and translational activity. The sedimentation gradient is indicated by a horizontal arrow, and absorbance at 254 nm is plotted on the *y*-axis to visualize ribosomal RNA content. Peaks corresponding to the 40S and 60S ribosomal subunits, monosomes (80S), and polysomes are annotated, along with half-mer formations. Insets display the polysome-to-monosome (P/M) ratio and the 40S/60S subunit ratio, providing quantitative measures of translational efficiency and ribosomal subunit balance.

Our previous studies demonstrated that treatment with TEMPO significantly affects ribosome biogenesis in budding yeast, as revealed by RNA-Seq analysis [18]. In the present study, we confirmed that 4-hydroxy-TEMPO similarly alters the expression of ribosomal protein genes, although the extent of its impact appears to be more moderate. Given the central role of ribosome biogenesis in cellular homeostasis and protein synthesis, we next performed polysome profiling to determine whether the observed transcriptional changes translate into functional alterations in ribosome assembly and activity. In control conditions (Figure 10A), cells exhibit a typical polysome distribution with clearly defined peaks for 40S, 60S, and 80S ribosomal subunits and a robust polysome region, indicative of active translation (P/M = 4.21 ± 0.008; 40/60 = 0.79 ± 0.006). Treatment with 1 mM hydrogen peroxide for 1 h (Figure 10B) results in a pronounced collapse of polysomes, reflected by a sharp decrease in the P/M ratio (0.2 ± 0.005) and an increased 40/60 ratio (1.2 ± 0.047), suggesting impaired initiation and accumulation of free 40S subunits due to oxidative damage. Similarly, exposure to 10 mM TEMPO for 2 h (Figure 10C) leads to severe translational repression (P/M = 0.15 ± 0.006; 40/60 = 0.79 ± 0.012), indicating that despite its antioxidant classification, high-dose TEMPO exerts cytotoxic effects that disrupt ribosome function. In contrast, treatment with 10 mM 4-hydroxy-TEMPO for 2 h (Figure 10D) shows partial restoration of polysome formation (P/M = 3.84 ± 0.025; 40/60 = 1.09 ± 0.027), suggesting that this compound more effectively preserves translational capacity. Polysome profiling at 10 mM 4-hydroxy-TEMPO (2 h) thus qualitatively supports a milder impact of this nitroxide on translation compared with H_2_O_2_ and TEMPO at high doses, as indicated by the partial preservation of the polysome region and a relatively high P/M ratio (Figure 10).

## 3. Discussion

4-Hydroxy-TEMPO is a stable nitroxide radical recognized for its potent antioxidant and redox-modulating activities in biological systems. Its unique ability to reversibly cycle between nitroxide, hydroxylamine, and oxoammonium states enables it to function as a SOD mimetic and an efficient scavenger of ROS and RNS [8,22]. Although 4-hydroxy-TEMPO exhibits promising biochemical properties, current knowledge regarding its cellular effects remains limited, with relatively few studies exploring its mechanisms of action or therapeutic potential in biological systems.

In this study, we employed the budding yeast *S. cerevisiae* as a genetically tractable and evolutionarily conserved model organism to explore the cellular impact of 4-hydroxy-TEMPO. Yeast offers a powerful system for dissecting fundamental biological processes, including oxidative stress responses, autophagy, and programmed cell death, which are highly relevant to human health and disease [23]. A recent study by Dobosz et al. demonstrated that 4-hydroxy-TEMPO acts in yeast cells as an effective but slowly penetrating antioxidant. After entering the cells, it neutralizes free radicals through reduction. Due to its hydrophilic nature, it acts more slowly than TEMPO but is less cytotoxic, confirming its potential use as a mild antioxidant in biological models [24]. In this study, we employed WT yeast alongside isogenic deletion mutants—*sod1Δ*, *sod2Δ*, *yap1Δ*, and *rad52Δ*—to dissect the molecular mechanisms underlying the cellular response to 4-hydroxy-TEMPO. *SOD1* and *SOD2* encode cytosolic and mitochondrial SOD, respectively, which are critical for detoxifying superoxide radicals [25,26]. *YAP1* encodes a transcription factor central to the oxidative stress response [27], while *RAD52* is essential for homologous recombination and DNA repair [28]. These mutants were selected to evaluate the compound’s impact on redox homeostasis and genome stability—key determinants of cellular aging. Our recent studies using TEMPO demonstrated that this nitroxide compound arrests the cell cycle and plays a critical role in modulating chronological aging in the yeast model [18]. Building on these findings, we aimed to investigate the physiological effects of 4-hydroxy-TEMPO, including growth rate, cell cycle progression, genotoxicity, and transcriptomic changes via RNA-Seq analysis. Notably, to date, no data have been reported on the effects of 4-hydroxy-TEMPO in yeast cells. Here, we show that this nitroxide significantly impairs yeast cell growth in a concentration-dependent manner, with the most pronounced effects observed in *sod1Δ* and *sod2Δ* mutants. Interestingly, while TEMPO exhibited similar phenotypic effects, 4-hydroxy-TEMPO required substantially higher concentrations to elicit comparable responses. Altered growth kinetics were strongly associated with cell cycle disturbances, particularly the accumulation of cells in the G1 phase.

Aging is commonly understood as a gradual and cumulative decline in an organism’s ability to maintain homeostasis, repair cellular damage, and adapt to environmental stressors [29]. In yeast, aging can be studied through two complementary models: replicative aging, which tracks the number of daughter cells produced by a single mother cell, and chronological aging, which measures the survival of non-budding cells in a stationary phase [30,31,32]. Chronological aging is especially relevant for understanding aging in post-mitotic cells, such as neurons, and is widely used to study stress resistance, metabolic regulation, and cell death mechanisms [33]. Given the universal desire to extend healthy lifespan, identifying factors that delay aging is of fundamental importance. Yeast models provide a simplified yet informative system to uncover genetic and chemical modulators of aging, offering insights that may be translatable to higher eukaryotes. In this study, we found that 4-hydroxy-TEMPO delays chronological aging in WT, *rad52Δ*, and *yap1Δ* strains. In contrast, it had no effect on the aging rate of *sod2Δ* cells and significantly accelerated aging in *sod1Δ* mutants. Our findings reveal that 4-hydroxy-TEMPO exerts a hormetic effect on yeast cells, characterized by a biphasic dose–response. At lower concentrations, the compound delays chronological aging in wild-type, rad52Δ, and *yap1Δ* strains, likely by inducing a mild oxidative stress that activates adaptive stress response pathways. This is consistent with the concept of hormesis, wherein low-level exposure to a stressor enhances cellular resilience and longevity through the upregulation of protective mechanisms [34,35,36,37,38]. A limitation of the present study is that regrowth kinetics were not directly tested using washout or serial passaging assays; therefore, the regrowth-like feature observed in CLS curves should be interpreted descriptively rather than mechanistically.

However, this beneficial effect is not universal. In *sod1Δ* mutants, which lack cytosolic SOD and are therefore highly susceptible to oxidative damage, the same concentrations of 4-hydroxy-TEMPO that were protective in other strains led to accelerated chronological aging. This suggests that in the absence of key antioxidant defenses, even low doses of redox-active compounds may exceed the threshold for beneficial stress and instead trigger cytotoxic effects. These results underscore the importance of cellular redox buffering capacity in determining the outcome of hormetic interventions and highlight the context-dependent nature of nitroxide-based therapeutics. The concentrations used in CLS experiments were intentionally higher than those applied in short-term assays. Short-term experiments (growth curves, cell-cycle analysis, ROS, FUN-1) were designed to capture early cellular responses and therefore used moderate doses (3–5 mM) or, in the case of transcriptomic profiling, a higher dose (20 mM) to maximize the transcriptional response. In contrast, CLS requires monitoring survival over several weeks; thus, a strain-specific titration was necessary to identify sub-toxic concentrations that modulated lifespan without inducing early death. More sensitive mutants (*sod1Δ*, *sod2Δ*) tolerated only low doses, whereas WT and *yap1Δ* strains required higher concentrations (up to 20 mM) to elicit measurable effects. Importantly, CLS comparisons were always made within the same strain, ensuring that dose differences do not affect the interpretation of lifespan changes. Instead, they reflect the specific interaction between 4-hydroxy-TEMPO and the physiological state of the cells during aging. We note, however, that future studies should complement PI-based CLS measurements with direct quantification of pH and acetic acid levels in aging cultures to more precisely assess the contribution of medium acidification to lifespan modulation.

Transcriptomic profiling of *S. cerevisiae* treated with 4-hydroxy-TEMPO revealed extensive reprogramming of gene expression, with significant enrichment of pathways related to ribosome biogenesis and oxidative phosphorylation. Notably, genes encoding ribosomal proteins were upregulated, indicating an activation of the translational machinery in response to nitroxide exposure. Paradoxically, this transcriptional response was accompanied by a marked growth delay in wild-type yeast cells, suggesting that increased ribosomal gene expression does not translate into enhanced proliferation. The transcriptional response of ribosome-related genes under 20 mM 4-hydroxy-TEMPO does not match the canonical ESR pattern, where most RP/Ribi genes are uniformly repressed. Instead, we observe a heterogeneous, redox-dependent reorganization of ribosome-associated gene expression, with many RP genes upregulated and a subset of mitochondrial and translation-associated factors downregulated. We propose that the upregulation of many RP genes under 4-hydroxy-TEMPO represents a compensatory or rebalancing response of the translational apparatus in a redox-stressed cell, in which initiation or ribosome engagement is limited (monosome accumulation), prompting selective transcriptional upregulation of ribosomal components rather than global Ribi shutdown as in the canonical ESR. Flow cytometry analysis further demonstrated that 4-hydroxy-TEMPO treatment led to accumulation of cells with 1C (G1 phase) DNA content, pointing to a disruption in the coordination between growth signals and cell cycle progression. S phase fractions should be interpreted cautiously and DNA content analysis alone cannot unambiguously identify the exact molecular checkpoint; therefore we use these data primarily to support the broader conclusion that 4 hydroxy-TEMPO perturbs cell cycle progression in a dose and genotype-dependent manner. Our findings suggest that the cellular response to 4-hydroxy-TEMPO involves a complex interplay between redox signaling, translational control, and cell cycle regulation. Similar transcriptomic and phenotypic effects were observed following treatment with TEMPO, suggesting that nitroxide radicals broadly influence cellular homeostasis [18]. The RT-qPCR validation further refines our view of the transcriptional response to 4-hydroxy-TEMPO. Up-regulation of SOD1 confirms activation of antioxidant defenses and supports the notion that the compound primarily acts as a redox modulator rather than a purely cytotoxic oxidant. The increase in RPS24A expression is particularly noteworthy, as ribosomal protein genes are typically repressed during the canonical environmental stress response; its induction therefore reinforces the idea that 4-hydroxy-TEMPO elicits a non-canonical, redox-driven program of translational remodeling. In contrast, the significant down-regulation of HSP104 indicates that, despite redox activation, cells do not mount a full proteotoxic or heat-shock response, in line with our observation that polysomes are only partially affected and that growth inhibition is moderate. Together, these qPCR results link the global RNA-Seq patterns to specific functional markers and strengthen the conclusion that 4-hydroxy-TEMPO triggers a distinct combination of antioxidant activation, selective support of the translational machinery and limited induction of classical chaperone networks. To further investigate their impact on translation, we performed polysome profiling, which revealed distinct alterations in ribosome biogenesis, particularly at the level of monosomes. Both TEMPO and 4-hydroxy-TEMPO treatments led to a pronounced accumulation of ribosomal monosomes, a pattern reminiscent of the cellular response to hydrogen peroxide-induced oxidative stress. The enrichment of monosomes may reflect a bottleneck in translation initiation or a stress-induced shift toward translational repression, despite transcriptional upregulation of ribosomal genes. This phenomenon suggests that nitroxide exposure disrupts the balance between ribosome production and functional engagement in translation, contributing to cell cycle arrest and reduced proliferation. A limitation of the present study is that polysome profiling was performed at 10 mM 4-hydroxy-TEMPO, whereas transcriptomic profiling was conducted at 20 mM. Therefore, the polysome data should be viewed as qualitative support for a distinct translational response to 4-hydroxy-TEMPO at high dose, rather than as a direct functional readout of the exact RNA-Seq condition. Future work will be needed to obtain polysome profiles under the precise RNA-Seq exposure conditions to more tightly connect transcriptomic remodeling with ribosome engagement.

The present work employs budding yeast *S. cerevisiae* as an experimental system, a model that has proven highly valuable for uncovering conserved principles governing cellular aging, redox regulation, and stress adaptation [19,39]. At the same time, several limitations inherent to this model must be acknowledged when considering the broader biological and translational implications of the findings. A fundamental constraint arises from the unicellular nature of yeast. Consequently, physiological strategies that favor survival at the expense of proliferation in yeast may not translate directly to mammalian contexts, where cell-cycle control, differentiation, and tissue homeostasis are tightly interconnected [29]. In addition, the transcriptional and physiological changes induced by 4-hydroxy-TEMPO in this study are most consistent with a shift toward a stress-adapted, maintenance-oriented cellular state rather than an enhancement of biosynthetic or proliferative capacity. Moreover, mitochondrial function, redox buffering capacity, and cell-cycle checkpoints are regulated in a more complex and context-dependent manner in higher eukaryotes [8,40,41,42].

Taken together, while yeast offers a powerful platform for defining fundamental redox-dependent regulatory strategies, the extrapolation of these findings to human biology should be approached with caution.

## 4. Materials and Methods

### 4.1. Preparation of 4-Hydroxy-TEMPO Solutions

4-hydroxy-TEMPO was handled as a light-sensitive redox-active nitroxide. Aqueous stock solutions were prepared freshly by dissolving the compound in ultrapure water to obtain a concentrated stock, followed by vortexing until complete dissolution. Stocks were protected from light and stored at 4 °C. To limit slow redox interconversion between the nitroxide, hydroxylamine, and oxoammonium forms, stock solutions were freshly prepared and used immediately. Working solutions were prepared immediately prior to each experiment by diluting the aqueous stock directly into the appropriate medium (YPD or SDC, or buffer as specified) to achieve the final concentrations used in each assay (typically 3–5 mM for short-term assays and up to 20 mM for selected experiments, as indicated in the respective Methods sections). Final solutions were mixed gently, kept protected from light, and used within the same experimental session. Under these conditions, no visible precipitation was observed, and the experimental outcomes were reproducible across independent biological replicates.

### 4.2. Yeast Strains and Culture Conditions

The yeast strains employed in this study are detailed in Table 2. Cultures were grown either in liquid YPD medium (composed of 1% Difco yeast extract, 1% Bacto peptone, and 2% glucose) with shaking at 150 rpm, or on solid YPD agar plates containing 2% agar. Chronological aging assay was assessed in synthetic defined complete (SDC) medium containing 2% (*w*/*v*) glucose and supplemented with the required amino acids and uracil. Unless otherwise stated, all experiments were conducted at 28 °C.

### 4.3. Assessment of Yeast Growth Dynamics

To assess the growth rate, cultures were initiated from a single colony and grown in liquid YPD medium, either supplemented (5–20 mM) or not with 4-hydroxy-TEMPO (Sigma-Aldrich, St. Louis, MO, USA). Cultures were incubated for 12 h at 28 °C with shaking at 1200 rpm using a Heidolph Incubator 1000 (Heidolph Instruments GmbH & Co. KG, Schwabach, Germany) (in 96-well microplates). Optical density at 600 nm (OD_600_) was measured every 2 h over the 12 h period using an Anthos 2010 microplate reader (Type 17550, Biochrom Ltd., Cambridge, UK).

### 4.4. Spot Assay

Yeast cells were grown overnight to reach the exponential phase and subsequently serially diluted to defined cell concentrations. Five microliters of each dilution were spotted onto YPD agar plates containing varying concentrations of 4-hydroxy-TEMPO (5–15 mM). Plates were incubated at 28 °C, and growth was evaluated after 48 h. All phenotypic observations were confirmed in three independent biological replicates.

### 4.5. Evaluation of Cell-Cycle Distribution

Analysis of cell cycle progression was carried out using the Muse™ Guava Cell Analyzer (Cytek Biosciences, Fremont, CA, USA). Following treatment with 4-hydroxy- TEMPO or control, yeast cells were washed with phosphate-buffered saline (PBS), collected, resuspended in PBS, and fixed in ice-cold 70% ethanol. Exponentially growing BY4741 and mutant strains were treated with 3 mM or 5 mM 4-hydroxy-TEMPO for 2 h before ethanol fixation and Muse™ Cell Cycle staining. Fixed samples were stored at −20 °C for 24 h. Ethanol-fixed cells were washed with PBS and stained with Muse™ Cell Cycle Reagent (MCH100106, Luminex, Austin, TX, USA), which contains propidium iodide and RNase A in a single solution, according to the manufacturer’s instructions. Samples were then analyzed using the Muse flow cytometer (Cytek Biosciences, Fremont, CA, USA). Cell cycle phase distribution—G0/G1, S, and G2/M—was determined based on DNA content histograms.

### 4.6. Cell Budding Ability

To assess the budding capacity of yeast cells, 20 µL of each cell suspension was spotted onto solid YPD agar plates (without or with 4-hydroxy-TEMPO (0–20 mM)). Microscopic images were captured at the start of the experiment and at subsequent time points (0 h, 3 h, 6 h, 12 h, and 24 h) using a Nikon Eclipse E200 microscope (Nikon Corporation, Tokyo, Japan) equipped with an Olympus DP26 digital camera (Olympus Corporation, Tokyo, Japan). This approach enabled visual monitoring of budding dynamics over time under different growth conditions.

### 4.7. Chronological Lifespan (CLS) Assay

Chronological lifespan was assessed in SDC medium containing 2% (*w*/*v*) glucose and supplemented with the required amino acids and uracil [19]. Viability was monitored at multiple time points (days 2, 4, 7, 14, 21, 28, and 35) using propidium iodide (PI) (5 μg/mL) staining to quantify cell survival. Data represent mean values from at least three independent experiments. The concentrations of 4-hydroxy-TEMPO used in CLS assays (5–20 mM) were selected based on preliminary titration experiments performed separately for each strain. For every genotype, the highest concentration that did not induce acute cytotoxicity during exponential growth, yet produced a measurable alteration of the survival curve, was chosen. This approach ensured that CLS measurements reflected long-term aging effects rather than immediate lethality. Because all CLS assays were performed in SDC medium under strictly identical conditions, differences between treated and untreated cultures within a given strain cannot be attributed to medium composition alone but instead reflect the interaction between 4-hydroxy-TEMPO and the physiological state of aging cells. 4-hydroxy-TEMPO remained present in the cultures throughout the chronological lifespan experiment; viability was assessed at defined time points without a deliberate compound-withdrawal step.

### 4.8. Cell Viability Assessment Using Propidium Iodide Staining

Cell death (during CLS assay) was evaluated by staining with propidium iodide (PI). Yeast cells were resuspended in PBS and incubated with 5 μg/mL PI (Sigma-Aldrich, St. Louis, MO, USA) for 15 min in the dark at 21 °C, following previously described protocols. Fluorescence images were captured using an Olympus BX51 fluorescence microscope (Tokyo, Japan) equipped with a DP-72 digital camera and cellSens Dimension software (Version 4.4, Evident Scientific/Olympus Corporation, Tokyo, Japan). Dead cells were visualized in the red fluorescence channel (λ_ex_ = 535 nm; λ_em_ = 617 nm). Data represent mean values from three independent biological replicates.

### 4.9. Measurement of Cellular Metabolic Activity

Exponentially growing cells were pre-incubated with 3 mM or 5 mM 4-hydroxy-TEMPO (or left untreated) for 2 h at 28 °C before staining with FUN-1. Metabolic activity was quantified using the FUN-1 fluorescent probe (Molecular Probes, Eugene, OR, USA), following the manufacturer’s instructions. Yeast cells were incubated with FUN-1 for 15 min at 28 °C in the dark. Fluorescence was measured using a Tecan Infinite 200 microplate reader at λ_ex_ = 480 nm and λ_em_ = 500–650 nm. Metabolic activity was expressed as the ratio of red (λ = 575 nm) to green fluorescence (λ = 535 nm). Results are presented as mean ± standard deviation (SD) from at least four independent cultures per strain.

### 4.10. Quantification of Superoxide Anion Production in Yeast Cells

Cells were treated with 3 mM or 5 mM 4-hydroxy-TEMPO for 2 h, or with 1 mM H_2_O_2_ (Sigma-Aldrich, St. Louis, MO, USA) for 2 h (positive control), prior to DHE (Sigma-Aldrich, St. Louis, MO, USA) loading. Superoxide anion generation, a key reactive oxygen species (ROS), was quantified using dihydroethidium (DHET; final concentration 18.9 μM; Invitrogen, ThermoFisher Scientific, Waltham, MA, USA), following established protocols [43]. Yeast cells in exponential growth phase were rinsed with sterile water and adjusted to a final concentration of 1 × 10^8^ cells/mL in 100 mM phosphate buffer (pH 7.0) supplemented with 0.1% (*w*/*v*) glucose and 1 mM sodium EDTA. Dihydroethidium (DHE) fluorescence was monitored using the TECAN Infinite 200 microplate reader (Tecan Trading AG, Männedorf, Switzerland) at an excitation wavelength of 518 nm and emission at 605 nm, maintained at 28 °C. All basic chemicals (phosphate salts, glucose, EDTA) were purchased from Sigma-Aldrich (St. Louis, MO, USA). Under these conditions, the signal reflects the combined fluorescence of DHE oxidation products (2-hydroxyethidium and ethidium) intercalated into DNA and thus serves as a global indicator of oxidative activity/redox status rather than a specific quantitative readout of superoxide levels. Results are presented as mean values from three independent replicates.

### 4.11. RNA Sequencing and Transcriptomic Analysis

For transcriptomic analysis, exponentially growing BY4741 cells were used. Cultures were treated with 20 mM 4-hydroxy-TEMPO for 2 h prior to RNA extraction, and untreated BY4741 cultures served as controls. Total RNA was extracted using the Yeast RiboPure RNA Purification Kit (AM1926, Invitrogen, Waltham, MA, USA), following the manufacturer’s protocol. Approximately 2 × 10^7^ freshly grown cells were harvested after overnight incubation. RNA quality and concentration were assessed using a Tecan Infinite 200 microplate reader (Tecan Group Ltd., Männedorf, Switzerland). Aliquots of ~5 mg RNA were stored at −80 °C at a concentration of 300 ng/μL.

SortMeRNA analysis indicated that rRNA-derived reads constituted <1.5% of total reads in all libraries, confirming low rRNA contamination and high RNA quality. RNA libraries were prepared using the MGIEasy RNA Library Prep Set MGI Tech Co., Ltd. (Shenzhen, China) and sequenced using DNBSeq technology on the BGISEQ-500 platform (BGI, Hong Kong, China). Transcriptomic data represent the average of three independent experiments.

Raw sequencing data were processed using the DESeq2 package in R. Gene Ontology (GO) annotations were retrieved using the BioMart data mining tool (Ensembl release 115, European Bioinformatics Institute, Hinxton, UK) [44]. Enrichment analysis of GO terms was performed using the opGO package (version 2.52.0, R/Bioconductor). to identify biological processes significantly overrepresented among differentially expressed genes. All processed RNA-Seq data, including normalized counts and DESeq2 statistical outputs, are available in Supplementary Dataset S1.

### 4.12. cDNA Synthesis and Quantitative Real-Time PCR Analysis

Total RNA extracted from yeast cells was divided into aliquots, dissolved in 15 µL of RNase-free water, and stored at −80 °C until use. Prior to cDNA synthesis, RNA concentration was measured using a Qubit 4 Fluorometer with the Qubit RNA High Sensitivity Assay Kit (Thermo Fisher Scientific, Waltham, MA, USA) according to the manufacturer’s protocol.

Complementary DNA (cDNA) was generated from 300 ng of total RNA using the smART RT-PCR Kit (EURx, Gdańsk, Poland) in a final reaction volume of 20 µL. Reverse transcription was performed following a two-step procedure recommended by the manufacturer. In the first step, 10.5 µL of diluted RNA was combined with Oligo (dT) primers, random hexamers, and dNTPs. In the second step, 5× cDNA synthesis buffer, DTT, RNase inhibitor, and the reverse transcriptase enzyme were added. The final cDNA products were stored at −20 °C until further analysis. Before qPCR, cDNA samples were diluted 1:2 to obtain a working concentration of approximately 150 ng/µL.

Quantitative real-time PCR (qPCR) was used to quantify the expression of selected genes using *ACT1* as the internal reference. The primer sequences were as follows: *ACT1* forward 5′-CGTTCCAATTTACGCTGGTT-3′ and reverse 5′-AGCGGTTTGCATTTCTTGTT-3′; SOD1 forward 5′-AGTGTTAAAGGGTGATGCC-3′ and reverse 5′-CACCGACATGTCTGACTTC-3′; RPS24A forward 5′-ATGTCTCCAAGGATGAATTGC-3′ and reverse 5′-CAGAGTTGTAGACCAAACCG-3′; and HSP104 forward 5′-GGCCATCAAGCAACAAGCTC-3′ and reverse 5′-GCGGTCTTACCGATACCTGG-3′. All primers were used at a final concentration of 450 nM.

Amplification reactions were carried out on a QuantStudio Real-Time PCR System (Applied Biosystems, Waltham, MA, USA). The cycling conditions were as follows: initial incubation at 50 °C, denaturation at 95 °C, 45 cycles of: 94 °C for 15 s, annealing at 52 °C (*RPS24A*, *SOD1*) or 54 °C (*HSP104*) for 30 s, and extension at 72 °C for 30 s.

A melting curve analysis was performed after amplification to confirm amplicon specificity. All qPCR reactions were run in triplicate. PCR product specificity was also confirmed by electrophoresis on 1.5% agarose gels.

To account for inter-plate variability, a calibrator cDNA sample was prepared by pooling equal amounts of cDNA from several representative samples. This calibrator was aliquoted and included on each qPCR plate (Applied Biosystems, Waltham, MA, USA) as an internal reference. The *ACT1* gene served as the endogenous normalization control.

Relative gene expression was calculated using the ΔΔCt method in QuantStudio Design and Analysis Software v1.5.2 (Applied Biosystems, Waltham, MA, USA). Expression values were reported as fold changes (RQ) relative to untreated controls.

### 4.13. Polysome Profile

Yeast BY4741 cells were exposed to 1 mM H_2_O_2_ for 1 h, 10 mM TEMPO for 2 h, or 10 mM 4-hydroxy-TEMPO for 2 h prior to polysome analysis. Polysome profile analyses were performed by centrifugation of total cell extracts in 10–50% linear sucrose gradients. Cell extracts were performed according to a previously described protocol [45]. Aliquots of cell lysate corresponding to 15 absorbance units at 260 nm (A260) were applied to a linear sucrose gradient and subjected to ultracentrifugation for 4.5 h at 84,865× *g* and 4 °C using an SW32Ti rotor (Beckman Coulter, Brea, CA, USA). Fractionated samples were subsequently analyzed with an ISCO Brendel Density Gradient Fractionator.

### 4.14. Statistical Analysis

All quantitative data are presented as mean ± SD from at least three independent biological replicates, unless otherwise indicated. Statistical significance was assessed using one-way analysis of variance (ANOVA) followed by Dunnett’s post hoc test to compare WT and isogenic strains under different growth conditions. A *p*-value < 0.05 was considered statistically significant. All statistical analyses were performed using Statistica software (version 13.3; StatSoft Inc., Tulsa, OK, USA).

## 5. Conclusions and Perspectives

Our study provides new insights into the biological activity of 4-hydroxy-TEMPO in budding yeast, highlighting its concentration-dependent effects on cell growth, cell-cycle progression, genome stability and aging. We show that 4-hydroxy-TEMPO induces substantial transcriptomic alterations that are accompanied by accumulation of cells with 1C/2C DNA content and impaired proliferation, indicating that nitroxide exposure affects fundamental cellular processes. Importantly, 4-hydroxy-TEMPO behaves differently from both TEMPO and classical oxidants. At high doses it is markedly less translationally toxic than TEMPO or H_2_O_2_, as evidenced by partial preservation of polysomes, despite strong transcriptional remodeling. RNA-Seq, supported by RT-qPCR, reveals a non-canonical, redox-driven stress program: classical ESR targets such as small heat-shock proteins and chaperones are induced, whereas many ribosomal protein genes are unexpectedly up-regulated rather than uniformly repressed. This atypical pattern, together with preserved polysome structure, points to a distinct mode of translation control under nitroxide-induced redox stress. We also demonstrate that 4-hydroxy-TEMPO exerts a clear hormetic effect on chronological aging, delaying aging in wild-type, *rad52Δ* and *yap1Δ* strains at low concentrations, while accelerating aging in *sod1Δ* mutants. This genotype-specific behavior underscores the critical role of redox buffering capacity in determining the outcome of nitroxide exposure. The qPCR data showing *SOD1* up-regulation and *HSP104* down-regulation are consistent with mild redox stress without severe proteotoxicity, further supporting this interpretation.

Taken together, our findings establish 4-hydroxy-TEMPO as a nitroxide with a previously uncharacterized combination of properties: reduced translational toxicity compared to TEMPO, non-canonical ESR-like transcriptional remodeling, hormetic and genotype-dependent effects on aging, and genoprotective activity. These features distinguish 4-hydroxy-TEMPO from other redox-active agents and suggest that it can serve as a useful tool for dissecting redox-dependent regulation of translation, metabolism and genome stability. Given the conserved nature of redox and ribosomal pathways, the mechanisms uncovered here may be relevant for understanding how nitroxide-based interventions modulate oxidative stress and aging in higher eukaryotes.

## Figures and Tables

**Table 1:** Functional enrichment analysis of transcripts altered in response to 4-hydroxy-TEMPO treatment. The table summarizes biological categories significantly affected following exposure to 4-hydroxy-TEMPO in WT yeast cells.

GO.ID	Term	Annotated	Significant	GO_Upregulated	GO_Downregulated
GO:0003735	structural constituent of ribosome	219	148	116	32
GO:0097177	mitochondrial ribosome binding	7	5	0	5
GO:0070181	small ribosomal subunit rRNA binding	9	6	4	2
GO:0070180	large ribosomal subunit rRNA binding	15	9	8	1
GO:0043022	ribosome binding	53	28	17	11
GO:0043023	ribosomal large subunit binding	8	5	4	1
GO:0043024	ribosomal small subunit binding	5	1	1	0
GO:0016723	oxidoreductase activity, acting on metal ions, NAD or NADP as acceptor	10	10	10	0
GO:0015453	oxidoreduction-driven active transmembrane transporter activity	19	16	0	16
GO:0016679	oxidoreductase activity, acting on diphenols and related substances as donors	9	9	0	9
GO:0016722	oxidoreductase activity, acting on metal ions	15	13	12	1
GO:0016491	oxidoreductase activity	319	176	92	84
GO:0016209	antioxidant activity	28	14	8	6
GO:0002181	cytoplasmic translation	168	121	113	8
GO:0006412	translation	456	273	177	96
GO:0006091	generation of precursor metabolites and energy	204	134	41	93
GO:0006812	monoatomic cation transport	194	127	74	53
GO:0000075	cell cycle checkpoint signaling	104	45	23	22
GO:0010948	negative regulation of cell cycle process	131	57	26	31
GO:0090068	positive regulation of cell cycle process	80	34	16	18
GO:0009060	aerobic respiration	88	58	10	48
GO:0022626	cytosolic ribosome	162	122	117	5
GO:0022625	cytosolic large ribosomal subunit	84	69	69	0
GO:0044391	ribosomal subunit	228	152	119	33
GO:0005840	ribosome	256	165	125	40
GO:0005886	plasma membrane	542	314	164	150
GO:0015934	large ribosomal subunit	132	91	73	18
GO:0071555	cell wall organization	232	118	61	57
GO:0008219	cell death	42	28	12	16

**Table 2:** Yeast Strains Used in the Experiment.

Strain	Genotype	Source
*BY4741*	MATa his3 leu2 met15 ura3	Euroscarf
*sod1Δ*	MATa his3 leu2 met15 ura3 YJR104C :: kanMX4	Euroscarf
*sod2Δ*	MATa his3 leu2 met15 ura3 YHR008C :: kanMX4	Euroscarf
*rad52Δ*	MATa his3 leu2 met15 ura3 YML032C :: kanMX4	Euroscarf
*yap1Δ*	MATa his3 leu2 met15 ura3 YML007W :: kanMX4	Euroscarf

## Data Availability

The original contributions presented in this study are included in the article/Supplementary Material. Further inquiries can be directed to the corresponding authors.

## Supplementary Materials

The following supporting information can be downloaded at: https://www.mdpi.com/article/10.3390/molecules31020376/s1, Figure S1: Clonogenic growth analysis of wild-type *Saccharomyces cerevisiae* and isogenic deletion mutants: *sod1Δ* (cytosolic superoxide dismutase), *sod2Δ* (mitochondrial superoxide dis-mutase), *rad52Δ* (DNA repair protein), and yap1Δ (oxidative stress-responsive transcription factor), treated and untreated with 4-hydroxy-TEMPO. Representative images were captured at 0, 3, 6, 12, 24, and 48 h to monitor colony formation dynamics. All experiments were performed in three independent biological replicates. Supplementary Dataset S1: Complete differential gene expression dataset for BY4741 cells treated with 20 mM 4-hydroxy-TEMPO for 2 h compared with untreated control cells. The file includes normalized counts, log2 fold-change values, standard errors, Wald statistics, raw p-values, and adjusted p-values (FDR) for all quantified transcripts. This dataset cor-responds to the RNA-Seq analyses presented in Figure 7, Figure 8 and Figure 9 and Table 2.
