# Supplementary material for: Nitroxide Hormesis in Yeast: 4-Hydroxy-TEMPO Modulates Aging, and Cell Cycle
Source: Molecules. 2026 Jan 21;31(2):376. doi: 10.3390/molecules31020376 (PMC12843841; doi:10.3390/molecules31020376)
Supplement: Supplementary file 1 [file molecules-31-00376-s001.zip › molecules-4073683-supplementary-Figure S1.pdf]

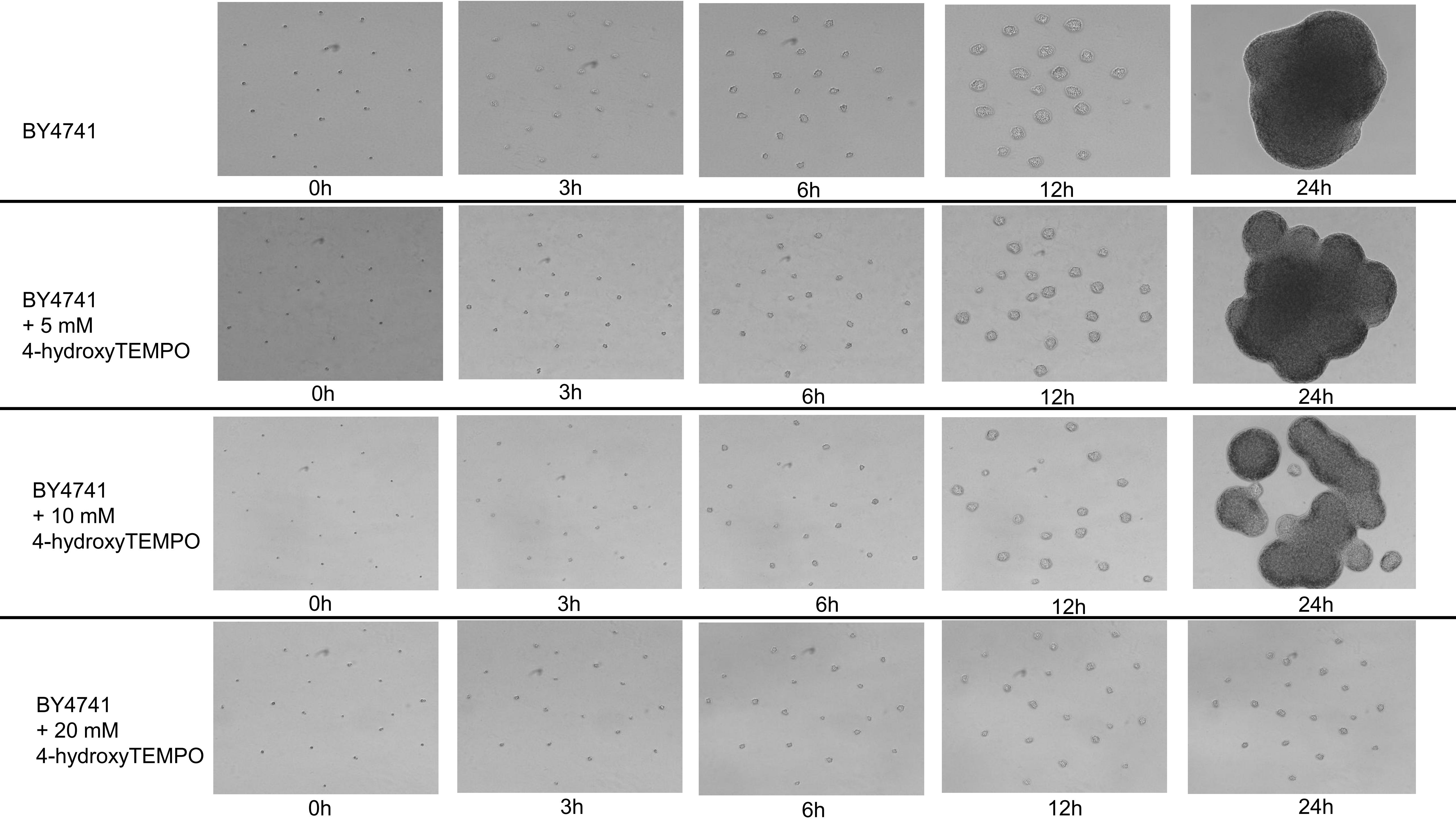
BY4741
0h
3h
6h
12h
24h
BY4741
+ 5 mM
4-hydroxyTEMPO
0h
3h
6h
12h
24h
BY4741
+ 10 mM
4-hydroxyTEMPO
0h
3h
6h
12h
24h
BY4741
+ 20 mM
4-hydroxyTEMPO
0h
3h
6h
12h
24h

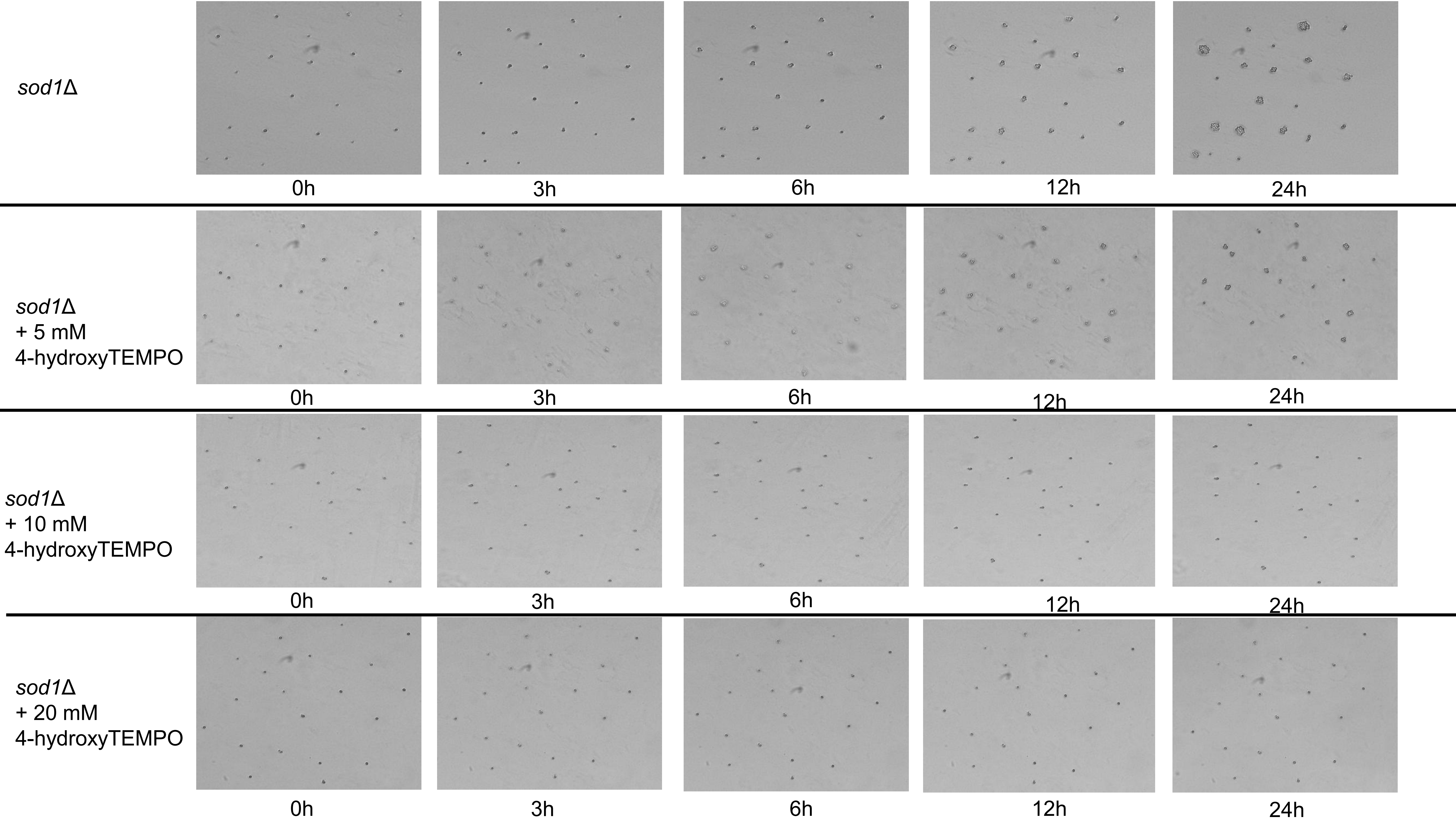
sod1Δ
0h
3h
6h
12h
24h
sod1Δ
+ 5 mM
4-hydroxyTEMPO
0h
3h
6h
12h
24h
sod1Δ
+ 10 mM
4-hydroxyTEMPO
0h
3h
6h
12h
24h
sod1Δ
+ 20 mM
4-hydroxyTEMPO
0h
3h
6h
12h
24h

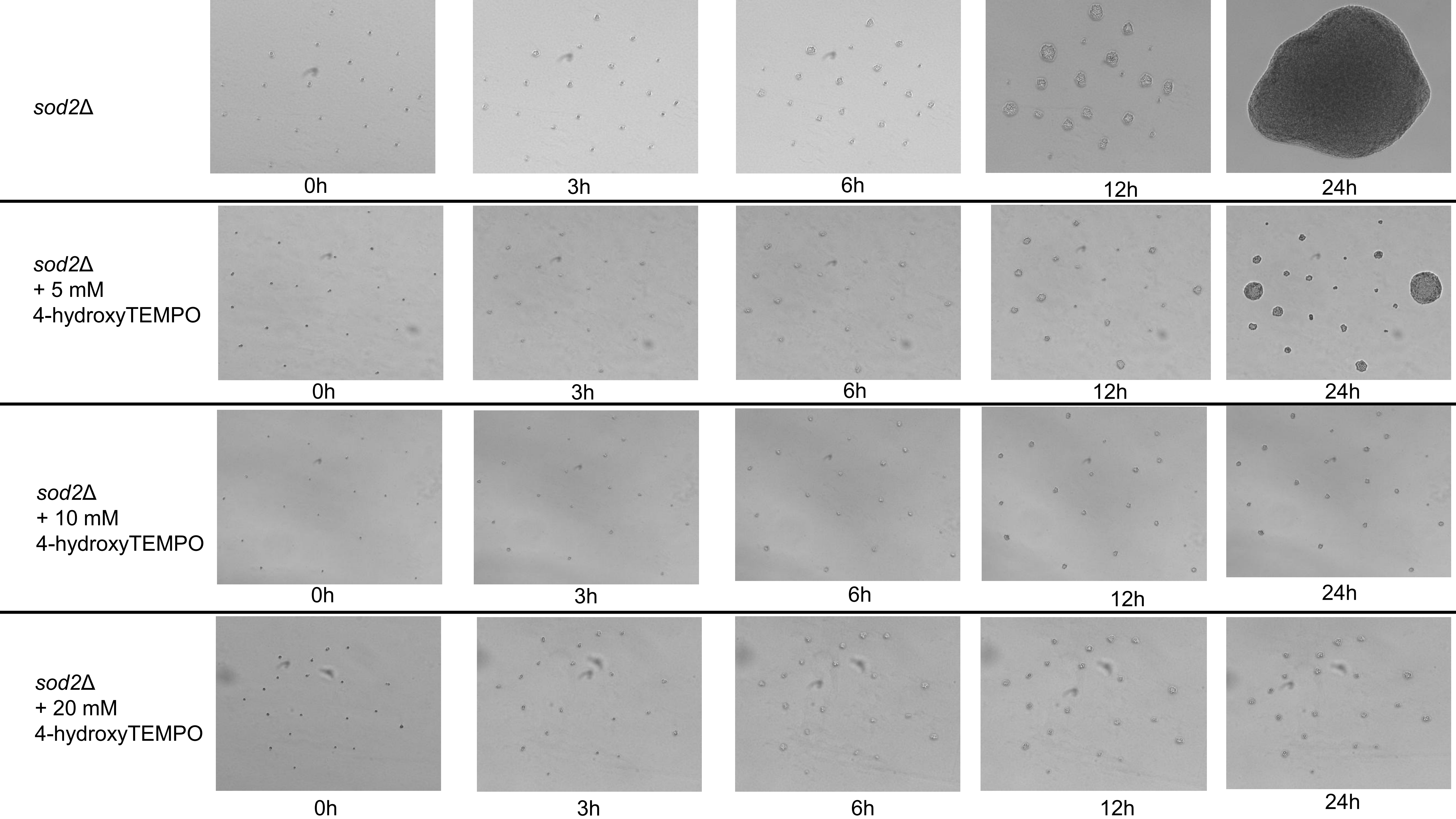
sod2Δ
0h
3h
6h
12h
24h
sod2Δ
+ 5 mM
4-hydroxyTEMPO
0h
3h
6h
12h
24h
sod2Δ
+ 10 mM
4-hydroxyTEMPO
0h
3h
6h
12h
24h
sod2Δ
+ 20 mM
4-hydroxyTEMPO
0h
3h
6h
12h
24h

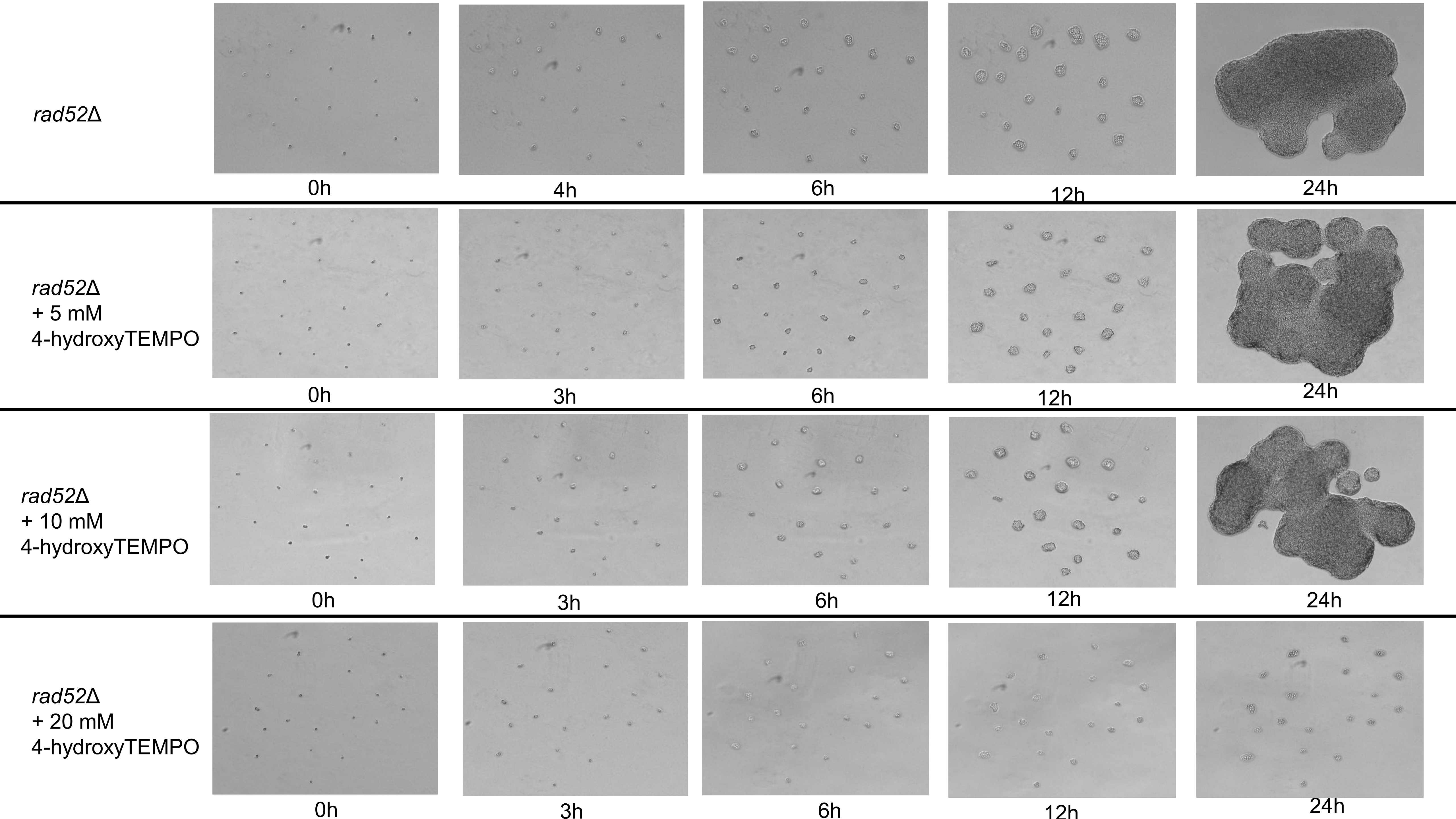

rad52Δ
0h
4h
6h
12h
24h
rad52Δ + 5 mM 4-hydroxyTEMPO
0h
3h
6h
12h
24h
rad52Δ + 10 mM 4-hydroxyTEMPO
0h
3h
6h
12h
24h
rad52Δ + 20 mM 4-hydroxyTEMPO
0h
3h
6h
12h
24h

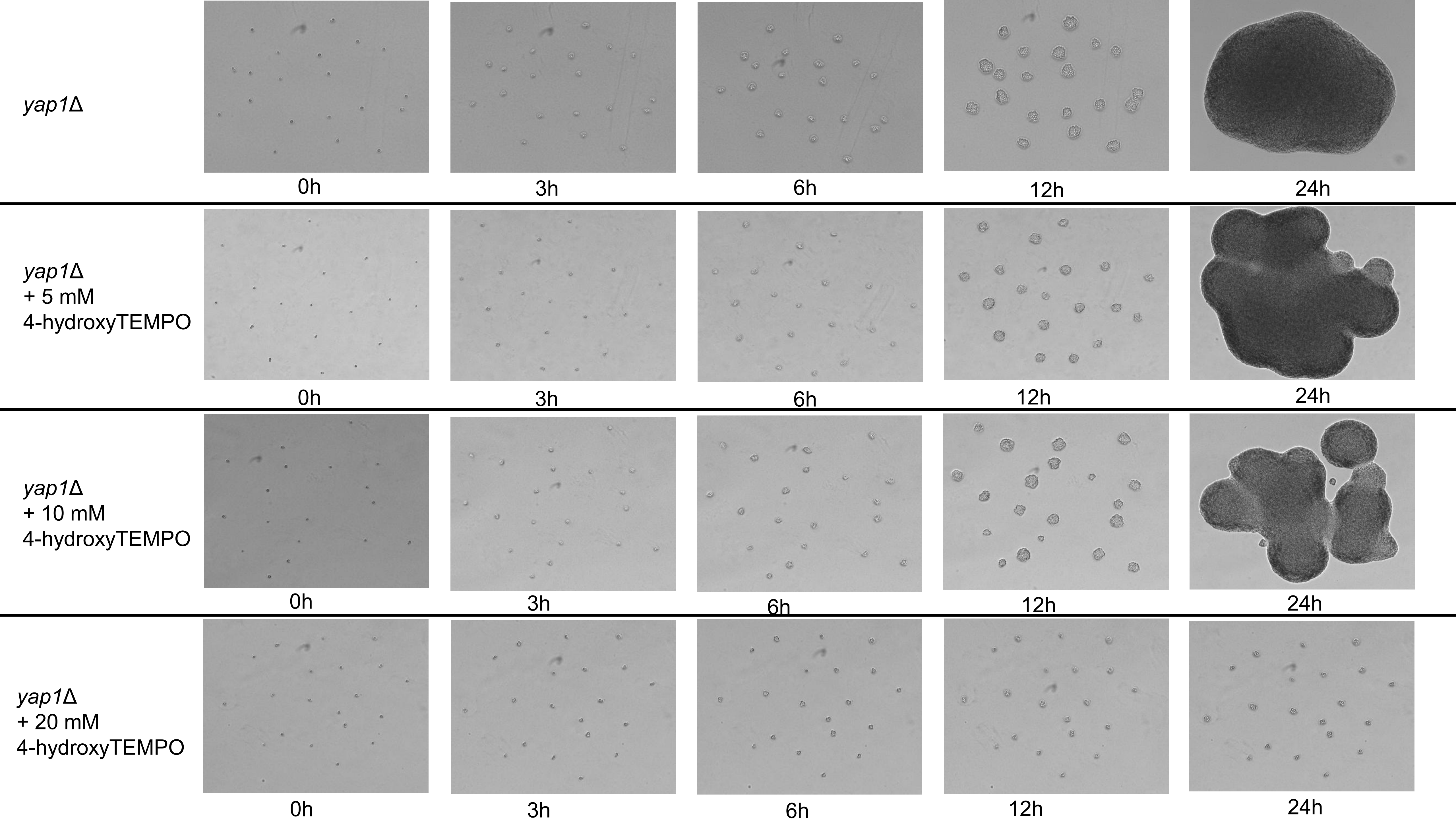
yap1Δ
0h
3h
6h
12h
24h
yap1Δ + 5 mM 4-hydroxyTEMPO
0h
3h
6h
12h
24h
yap1Δ + 10 mM 4-hydroxyTEMPO
0h
3h
6h
12h
24h
yap1Δ + 20 mM 4-hydroxyTEMPO
0h
3h
6h
12h
24h
